# Flexibility of representational states in working memory

**DOI:** 10.3389/fnhum.2014.00853

**Published:** 2014-11-06

**Authors:** Nahid Zokaei, Shen Ning, Sanjay Manohar, Eva Feredoes, Masud Husain

**Affiliations:** ^1^Department of Experimental Psychology, University of OxfordOxford, UK; ^2^Nuffield Department of Clinical Neurosciences, University of OxfordOxford, UK; ^3^School of Psychology and Clinical Language Sciences, University of ReadingReading, UK

**Keywords:** working memory, attention, representational states, retro cueing, incidental cueing

## Abstract

The relationship between working memory (WM) and attention is a highly interdependent one, with evidence that attention determines the state in which items in WM are retained. Through focusing of attention, an item might be held in a more prioritized state, commonly termed as the focus of attention (FOA). The remaining items, although still retrievable, are considered to be in a different representational state. One means to bring an item into the FOA is to use *retrospective cues* (“retro-cues”) which direct attention to one of the objects retained in WM. Alternatively, an item can enter a privileged state once attention is directed towards it through bottom-up influences (e.g., *recency effect*) or by performing an action on one of the retained items (*“incidental” cueing)*. In all these cases, the item in the FOA is recalled with better accuracy compared to the other items in WM. Far less is known about the nature of the other items in WM and whether they can be flexibly manipulated in and out of the FOA. We present data from three types of experiments as well as transcranial magnetic stimulation (TMS) to early visual cortex to manipulate the item inside FOA. Taken together, our results suggest that the context in which items are retained in WM matters. When an item remains behaviorally relevant, despite not being inside the FOA, re-focusing attention upon it can increase its recall precision. This suggests that a non-FOA item can be held in a state in which it can be later retrieved. However, if an item is rendered behaviorally unimportant because it is very unlikely to be probed, it cannot be brought back into the FOA, nor recalled with high precision. Under such conditions, some information appears to be irretrievably lost from WM. These findings, obtained from several different methods, demonstrate quite considerable flexibility with which items in WM can be represented *depending upon context*. They have important consequences for emerging state-dependent models of WM.

## Introduction

Working memory (WM) refers to the ability to hold and manipulate information in mind for brief periods of time (Baddeley, 2003). It has been proposed that not all information in WM is maintained in an equal state. For example, depending on task relevance such as likelihood of being probed, one item might require prioritization over others. These sorts of considerations have led some authors to argue that the state of WM representations might be determined by an interaction between long-term memory (LTM) and attention (e.g., Cowan, 1998; McElree, 1998; Oberauer, 2002).

**Figure 1:**
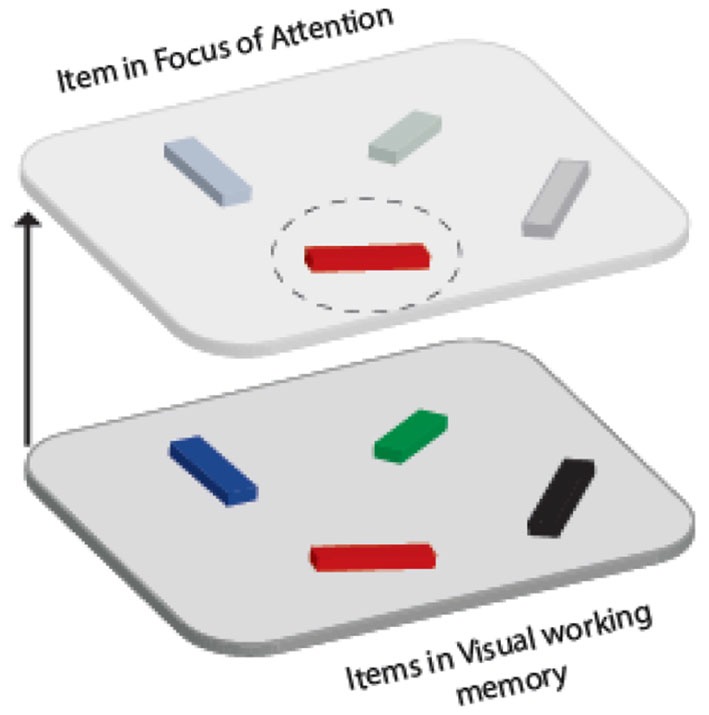
**Schematic representation of items maintained in visual WM in two different representational states**. Although multiple items can be maintained in WM (lower panel), one item (red object in top pane) might be held in a more prioritized state known as the “focus of attention” (FOA).

While these models differ in how the capacity and nature of representations in various states are determined (see Larocque et al., 2014 for a comprehensive review), they all agree on the existence of least two distinct states. One of these states has attention focused on an item (or a subset of items), rendering it in a more prioritized state so that it can be accessed more readily, with higher accuracy and/or fidelity (e.g., Lepsien and Nobre, 2007; Pertzov et al., 2012; Zokaei et al., 2014). In line with the most prominent theoretical models, we will refer to these prioritized items as being inside a focus of attention (FOA; as defined by Cowan, 1998; Oberauer, 2002, 2009). The remaining items outside FOA—although still retrievable—are considered to be in a different representational state (Figure 1).

The distinction between the items inside and outside FOA has been demonstrated both empirically (Lewis-Peacock et al., 2011; LaRocque et al., 2013; Stokes et al., 2013) and theoretically, often described in terms of the activated LTM proportion of short term memory (e.g., Oberauer, 2002). A substantial amount of research has focused on the qualities of representations of the item in FOA, but far less is known about the nature of the other items. One important but outstanding question that we address in this study is whether items can flexibly move in and out of FOA.

### Manipulation of representational states in WM

Behaviorally, the item in FOA is identified as the item that is recalled faster and more accurately compared to other retained items (see Nee and Jonides, 2013; Larocque et al., 2014 for examples). There are various methods for manipulating representational states in WM that result in such a behavioral advantage. In this section, we review selected studies that have used various approaches to shed light on the nature of the item in FOA and as a consequence on the other, non-focused items.

#### Retro-cues shift the focus of attention in WM

A commonly used means to bring an item into FOA is the use of retrospective cues (“retro-cues”) presented during the retention interval, with the intention of directing attention to one specific item in WM. Specifically, a retro-cue will indicate to the participant which item is highly likely to be probed for recognition or recall. A consequence of retro cues is higher accuracy or precision of recall for the cued item compared to other uncued or invalidly cued items (Griffin and Nobre, 2003; Lepsien and Nobre, 2007; Makovsik and Jiang, 2007; Sligte et al., 2008, 2010; Astle et al., 2009; Lepsien et al., 2011; Berryhill et al., 2012; Pertzov et al., 2012).

Some studies have further attempted to characterize the neural underpinnings of the item in FOA achieved through retro-cueing (e.g., Lepsien and Nobre, 2007; Harrison and Tong, 2009; Lewis-Peacock et al., 2011; Nelissen et al., 2013; Larocque et al., 2014). In one such investigation, participants were presented with a sequence of two images to remember from two different categories of faces and scenes (Lepsien and Nobre, 2007). After a retention period they were shown a retro-cue indicating the relevant item for the upcoming memory probe. Event-related functional magnetic resonance imaging (fMRI) demonstrated that orienting attention to one of the items during WM maintenance modulated the activity in region of the brain involved in perception of the cued item: fusiform face area for faces and parahippocampal place area for scenes.

Using multi-voxel pattern analysis (MVPA; e.g., Haxby et al., 2001; Haynes and Rees, 2006) some investigators have reported stimulus-specific patterns in early visual areas during WM retention for retro-cued items. In a landmark study Harrison and Tong (2009) demonstrated that the orientation of the Gabor patch retro-cued during WM maintenance (from two retained orientations) could be decoded from the activity patterns in visual areas V1-V4. Nelissen et al. (2013) employed a similar retro-cueing WM task and showed that cued items could be accurately decoded from occipitotemporal cortex, but that decoding was at chance level for the un-cued items (Nelissen et al., 2013; See also Lewis-Peacock et al., 2011).

From these findings one can conclude that the retro-cued item is maintained in a different state compared to non-cued items, with its maintenance dependent, at least to some extent, on early visual cortical regions known to be involved in perception of the maintained information.

#### Recency: last item in the focus of attention

Many investigations of WM present items serially rather than simultaneously. It has been known for a long time that the last item in a sequence is better recalled than previous ones: the recency effect. Some authors argue that the most recent item is in fact automatically in the FOA. Evidence comes from behavioral studies demonstrating retrieval advantage for the most recent item compared to items presented earlier in the sequence (McElree and Dosher, 1989, 1993; Neath, 1993; Hay et al., 2007; Blalock and Clegg, 2010). Furthermore, this last item is recalled faster, with more accuracy and precision compared to all previous items, with the magnitude of recency effect dependent on the number of previous items in the sequence (Gorgoraptis et al., 2011; Zokaei et al., 2011). Thus, the well-known recency effect in the WM literature might be due to the obligatory assignment of the last item to the FOA.

Brain imaging techniques have shed light on possible dissociation in neural correlates for maintenance of the last item compared to earlier items in a sequence. Using fMRI, Nee and Jonides (2008) investigated the probed-evoked neural signal of the most recently presented word compared to words presented earlier in a sequence. The recognition of the last item was accompanied by increased activation in the inferior temporal cortex. Similar findings have been presented in later studies (Nee and Jonides, 2011, 2013). Furthermore, recognition of the last word in the memory sequence was accompanied by less hippocampal activation when compared to recognition of all previous items in a sequence leading to intriguing suggestion that non-focused items are maintained by the hippocampus (Oztekin et al., 2009, 2010). Hence, there is both behavioral and neural evidence for dissociation of the most recent item into WM compared to previous items.

In addition recent findings using transcranial magnetic stimulation (TMS) have provided causal evidence for different representational states in visual WM (Zokaei et al., 2014). Two random dot kinematograms (RDKs) moving in different directions were presented sequentially, with the aim of bringing the second RDK into FOA. Following a delay period, participants had to recall the direction of one of the two previously-presented RDKs. Transcranial magnetic stimulation was administered during the delay of the WM task, prior to the presentation of the probe, to motion sensitive area MT+ which was hypothesized to be maintaining the remembered motion directions (Bisley and Pasternak, 2000; Pasternak and Greenlee, 2005). Transcranial magnetic stimulation impaired recall precision of the motion direction in FOA and, crucially, conversely improved precision for the other non-focused direction (Zokaei et al., 2014). These findings provide some of the first causal evidence that there are at least two representational states, with the maintenance of only the item in FOA relying on an area involved in its perception.

#### Incidental-cueing brings an item into focus of attention

A new method for manipulating representational states in WM has recently been developed; incidental cueing. This approach is different to the retro-cueing approach, because the cue is *not* explicitly predictive of upcoming memory recall. Instead, the rationale is that once an item from those maintained in WM is used for a cognitive operation that is incidental or orthogonal to the memory requirements of the task, that item will automatically enter the FOA.

In the study by Zokaei et al. (2014) participants were once again presented with two RDKs now simultaneously, above and below a fixation cross, and in two different colors. Participants were required to remember the directions of motion of each of the RDKs. During the delay period, the fixation cross changed to the color of one of the maintained motion directions and participants indicated with a key press the location of the motion direction of that color in the memory array, i.e., whether it had been above or below the fixation cross. The color of the fixation cross was not informative of the upcoming memory probe, and the judgment about the location of one of the RDKs was completely orthogonal to the requirement to remember the direction of motion of the RDKs. Nevertheless, adding this incidental task, resulted in higher precision of recall of the motion direction that also matched the color of the fixation cross. Thus, this item appeared to be in FOA simply by virtue of being “incidentally” cued. In agreement with the results from the TMS experiment described above, TMS applied here during the delay period, after the incidental cue, also disrupted recall of the cued item, and improved it for the non-cued item.

A similar rationale was employed by Lewis-Peacock et al. (2011) who used a dual response and cueing WM task adapted from Oberauer (2005). Following a delay after presentation of the memory array, a cue appeared highlighting the first item to be probed. After the response, a second retro-cue appeared that indicated either the same item as that previously probed or alternatively cued the participant to switch to the other item in WM. For this second cue, only the task-relevant item (the cued item) could be successfully decoded from the fMRI BOLD signal, whereas the irrelevant (non-cued) item could not. This occurred despite no behavioral loss in performance when the second cue was different to the first (Lewis-Peacock et al., 2011, see LaRocque et al., 2013 for electroencephalography (EEG) analog).

The findings reported in this section provide evidence for the existence of a FOA within WM representations: an item can be held in a more privileged state with its recall more accurate and with higher precision compared to all other items maintained in WM. But what about the other items in WM? What happens to them and how are they stored?

### The fate of items outside the focus of attention in WM

We now turn to investigations of how the items outside FOA are represented relative to those inside FOA. One hypothesis is that the item in FOA is protected from interference from un-cued items, with this protection coming at a cost for remembering un-cued items. Pertzov et al. (2012) simultaneously presented four oriented bars of different color and asked participants to remember their orientation. One of the bars was subsequently probed by its location or color. During the retention period, a retro-cue appeared indicating the item that was most likely to be later probed (70% validity). In the subsequent recall period, participants were asked to reproduce the exact orientation of one of the bars. Precision of recall was significantly worse for the invalidly cued trials (i.e., trials where one of the un-cued items were probed) compared to trials in which the probe was validly cued, and also to baseline trials in which there was no cue.

These results were interpreted as evidence for the validly cued item being in a state that was protected from interference by other items held in WM, with these other objects suffering from accelerated temporal decay. Similar findings have been reported by Lepsien and Nobre (2007) and Matsukura et al. (2007). In the latter study, participants were presented with two consecutive retro-cues, with the second cue 100% valid. The second cue could be same or different to the first. Two identical cues resulted in similar performance to a single valid retro-cue, while two different cues impaired performance. The authors explained their findings in terms of forgetting un-cued items due to either degradation or interference, alongside protection of the cued item.

Evidence for the degradation of un-cued items is, however, inconsistent. In WM change detection tasks, several studies report no significant impairment in recall of the un-cued information with two consecutive cues during the delay (Landman et al., 2003; Rerko and Oberauer, 2013). For example, Rerko and Oberauer (2013) presented participants with a memory array followed by either a single, two or three cues. The probed matched (50% trials) or mismatched the item that was cued last. There was no difference in accuracy between the cued and un-cued items.

The discrepancy in behavioral findings for memory of un-cued items may be explained by the degree of information conveyed by the cue. In tasks that show an effect on un-cued information (i.e., impaired performance in recalling those items) the cue carries predictive information indicating which will be the most relevant item—often the only one relevant item—for forthcoming memory recall. As a consequence, un-cued information is rendered behaviorally irrelevant because of the low probability that it will need to be accessed again. In effect, this therefore changes the task to WM for a single (cued) item only.

On the other hand, in studies that have failed to find an effect on un-cued items (i.e., recall/recognition accuracy is no different to cued items), participants did not need to attend to previous cues, but rather the last cue only (e.g., Landman et al., 2003; Rerko and Oberauer, 2013). Thus, only the final cue was informative with respect to the upcoming probe, and importantly with only 50% validity. It could therefore be argued that given the low amount of predictive information conveyed by the cue, it was rendered less effective compared to retro-cues with, for example, 70% validity. Moreover, in these studies the final cue was followed by a very short delay (<500 ms) prior to the presentation of the probe. But for a retro-cue to produce sufficient behavioral advantage, there should be at least 1 s between the presentation of the cue and the memory probe (Pertzov et al., 2012).

### Recall of items not in focus of attention

From the studies described above it is evident that research has mainly centered on the single, privileged item in FOA. As a consequence, far less is known about the nature of non-focused items, and whether these items can be brought back into FOA or are lost from memory. In light of previous findings, one can hypothesize that whether items can flexibly move in and out of the FOA might be highly dependent on their potential relevance to the task in hand. Thus, if a non-focused item has a high chance of being probed it might still be maintained in a state such that it can be retrieved with high quality.

We tested this hypothesis across four experiments in which we used a method for measuring WM performance that relies on participants to reproduce the exact qualities of the retained information, providing a measure of precision of recall (Gorgoraptis et al., 2011; Zokaei et al., 2011, 2013). Such sensitive measure of WM allows us to detect small changes in recall precision that may otherwise not be detectable with alternative measures. In the first two experiments we used cues that were orthogonal to the WM task at hand. We hypothesized that these cues would allow items to move flexibly in and out of the FOA, since they carry no information regarding the relevance of items in WM. On the other hand, in experiment 3 and 4, a retro-cue with 80% validity was used. Unlike the first two experiments, we predicted that in such a situation, non-focused (un-cued) items in WM might degrade to the extent that they cannot be brought back into FOA.

In our first two experiments we aimed to examine whether representational states of items in WM can flexibly change in situations in which *all items in WM remain potentially, behaviorally relevant throughout the trial*. In Experiment 1 we used two successive *incidental cues* while in Experiment 2, we used sequential presentation of items so that the last item was in FOA by virtue of *recency*. This was then followed by an incidental cue that could be same or different to the last item in the sequence. Note that this method of cueing always required participants to make a response, allowing us to confirm whether they attended to the cued item or not, rather than relying on participants to attend to the cue of their own volition, with no objective measure (e.g., Landman et al., 2003; Pertzov et al., 2012; Rerko and Oberauer, 2013).

## Experiment 1: effects of one or two incidental cues

### Methods

#### Participants

Twenty healthy individuals (13 male) with an average age of 27 (range: 19–35) participated. All had normal or corrected to normal vision and reported normal color vision. They provided written consent to the procedure of the experiment, which was approved by the local ethics committee.

#### Stimuli

On each trial, two RDKs were presented below and above the fixation cross, subtending 10° of visual angle. Each RDK consisted of 50 dots (0.1° visual angle each), displayed within an invisible circular aperture (5.7° of visual angle). The color of the top RDK was chosen at random on each trial to be either green or red, with the lower RDK assigned the other color (red or green).

Dot lifetime and density were constant during RDK presentation with 100% coherent motion (constant speed of 4.5°/s). Motion direction for each RDK was selected from 0–360° with no angular separation between the two motion directions on each trial. A mask consisting of 5000 dots (50% red), covering the entire screen was presented immediately after RDK offset.

Stimuli were displayed on 14.1” display (resolution 800 × 600 pixels, refresh rate 60 Hz). Participants were seated approximately 60 cm from the monitor in a dimly lit room.

#### Procedure

**Figure 2:**
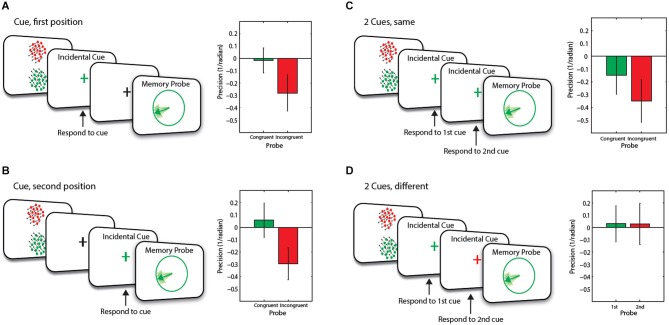
**Schematic and baseline corrected WM recall performance of trial events for Experiment 1**. Two items were presented in the memory array. This was then followed by **(A)** an incidental cue in the first position; **(B)** an incidental cue in the second position; **(C)** two identical consecutive incidental cues or **(D)** two different consecutive incidental cues, before the presentation of the memory probe. Error bars are SEM.

A schematic representation of the task and different conditions is illustrated in Figure 2. Each trial started with a fixation cross (500 ms), followed by the presentation of two RDKs (the memory array for 300 ms) and mask (100 ms). In this experiment, cues could be presented at two different time points, referred to as *1st position* and *2nd* *position* cues.

In the ***baseline condition*** (1/9th of trials and not shown in the figure), the RDKs were simply followed by 7.2 s blank interval before presentation of probe stimuli. In 4/9th of trials, the stimuli were followed by a blank delay (1 s) and then the fixation cross briefly changed to either red or green (100 ms) which served as our method of incidental cueing. Participants had to indicate with a key press as accurately and as fast as possible the location of the RDK (above or below fixation cross) that was the same color as the fixation cross.

On half these trials (i.e., 2/9th of the overall trials), the cue was followed by a 6.1 s blank interval before the presentation of the probe (***One cue, 1st condition***—Figure 2A). On the remainder of the trials (2/9th of the overall number of trials), the memory array was followed by a 4.1 s delay, then the incidental cue (100 ms) and another delay (3 s) before the presentation of the probe (***One cue, 2nd position***—Figure 2B).

In the remaining trials (4/9th of the overall trials), the cue was followed by 3 s delay before the presentation of a second incidental cue which could be the same (Figure 2C) or different color (Figure 2D) to the first cue (***Two cues condition***). Participants were informed that the cue was orthogonal to the memory task.

The memory probe was a circle (5.7° visual angle in diameter) presented at the center of the screen with a line from the center positioned at a randomly-selected orientation. On trials with one incidental cue and those with two identical colored cues, the color of the probe was the same (50% of trials—congruent) or different (incongruent) to the color of the incidental cue(s).

In trials with two differently colored cues, on half the trials the probe was same color as the first cue while on the remaining the trials it was presented in the same color as the second cue. Using a mouse, participants adjusted the orientation of the line within the circle until it matched the direction of motion of the probed RDK. Accuracy of this matching procedure was emphasized over response time. Participants completed a practice block (30 trials) followed by seven blocks of 36 trials, different conditions randomly intermixed within a block.

#### Precision calculation

Recall error for the memory for motion task was calculated as the difference in response angle from target angle (i.e., the actual angle of the probed item). Recall precision was defined as the reciprocal of standard deviation of response error (Philipp, 2009). Note that precision is a measure of variability with higher precision corresponding to lower variability in memory. Due to small number of trials for all experiments reported here, we were unable to perform mixture modeling (Bays et al., 2009) to dissect our the sources of error resulting in a specific pattern of performance.

### Results

Participant performed the *incidental cueing task* with a high level of accuracy over all conditions (see Table 1 for mean accuracy and response times for all conditions). Trials in which there were incorrect responses to incidental cues were excluded from analysis.

**Table 1:** **Mean accuracy and response times for the two cues for all conditions (accuracy in percentage and response times in ms)**.

Probe type	**1 cue, 1st position**		**1 cue, 2nd position**		**2 cues, same**		**2 cues, different**	
	Same	Different	Same	Different	Same	Different	1st item	2nd item
**Cue 1**								
**Accuracy (%)**	83	87	n/a	n/a	85	87	89	86
**RT (msec)**	742	730			715	723	730	722
**Cue 2**								
**Accuracy (%)**	n/a	n/a	86	84	86	85	89	88
**RT (msec)**			699	709	629	653	579	575

We examined* memory recall precision of motion direction* for congruent vs. incongruent trials, corrected by performance in the baseline (no cue) condition in conditions where only one memory item was incidentally cued (i.e., the 1cue conditions, and two cues of the same color). There was an overall significant main effect of congruency, of whether the memory probe was about the same or different item to that specified by the previous incidental cue (*F*_(1,19)_ = 11.06, *p* = 0.004). Thus recall precision was lower in the incongruent trials compared to congruent trials. This occurred in conditions when an incidental cue was presented in the first position (*t*_(19)_ = 3.7, *p* = 0.001, Figure 2A), or second position (marginal significance, *t*_(19)_ = 1.8, *p* = 0.084, Figure 2B) or when two cues of the same color were presented (*t*_(19)_ = 2.27, *p* = 0.035, Figure 2C). The incongruency effect for an incidental cue in the second position was marginally significant (*t*_(19)_ = 1.8, *p* = 0.084, Figure 2B). These findings indicate successful incidental cueing. Performing the incidental task (which is orthogonal to the motion direction memory task) made a systematic difference to recall precision on the memory task, depending on congruency of incidental cue to the RDK that was later probed.

Importantly, the cueing advantage was apparently driven by a decrease in recall precision on incongruent trials, compared to congruent trials. Compared to no-cue baseline condition (zero in figures), there were marginally significant decreases in recall precision on *incongruent* trials when one cue occupied the 1st position (*t*_(19)_ = 1.89, *p* = 0.07, Figure 2A) and when two cues of the same color were presented (*t*_(19)_ = 2.08, *p* = 0.051, Figure 2C). There was a significant drop in recall precision for *incongruent* trials when one cue occupied the 2nd position (*t*_(19)_ = 2.28, *p* = 0.035, Figure 2B).

The critical condition, however, was when participants were presented with a sequence of two *differently colored incidental cues*. Note that in this case, we can consider the data in terms of whether the probe was the same as the item specified by the first incidental cue, or whether it was the same as the second cue. Prior to the second cue, one item would be considered to be in FOA. After it, however, another item might be brought into the FOA. This now becomes the “focused” item. Importantly recall precision for this item was neither significantly different to the no-cue/baseline (*t*_(19)_ = 0.2, *p* = 0.85), nor to when a single cue was presented in 2nd position (*t*_(19)_ = 0.3, *p* = 0.8; Figure 2D).

This finding shows that relatively high quality information regarding “other” items in WM—at least, similar to the baseline state—can be retrieved, *in the context* of a task where participants know that they might be asked to switch their attention between items in WM. This occurred despite the fact that such items were recalled with lower precision when they had not been brought into the privileged state of FOA by incidental cueing (Figures 2A–C).

Precision of recall for the “defocused” item in this condition, that is the item specified by the first cue and considered to be in FOA prior to the second cue was also no different than baseline (*t*_(19)_ = 0.2, *p* = 0.83, Figure 2D) or to the item that was brought into FOA by the second cue (*t*_(19)_ = 0.02, *p* = 0.99, Figure 2D). The lack of an effect on this item is discussed below.

### Discussion

These results demonstrate that in situations where items outside FOA remain *potentially* *behaviorally relevant*, they can be brought into FOA. The no cue, baseline condition used here allowed us to examine whether the effect of incidental cueing on WM performance was due to improvement of the item in FOA, or a degradation of the item outside it. The findings presented here show that the incidental cueing advantage can be explained specifically by a decrease in recall precision for the item outside FOA—rather than an advantage for the focused item—compared to baseline. So performing a task that requires consideration of the item specified by the incidental cue leads to a degradation of memory of the other item in WM, but no simultaneous boost to the cued item. Thus incidental cueing appears to operate in a very specific manner on the contents of WM.

A second important issue in these experiments is the fate of the de-focused item, that is the item that was inside FOA before the participant’s attention was switched to another item as the trial progresses. Here, recall precision for the defocused item was comparable to baseline, as well as focused condition—but not worse. This might not be predicted on the basis of attention being drawn away from this item and instead raises the possibility that the capacity of FOA is larger than 1 item.

## Experiment 2: effects of incidental cue on recency

In the second experiment we aimed to examine whether representational states of items in WM can flexibly change in situations in which *all items in WM remain potentially, behaviorally relevant throughout the trial* using a different method. Here, we used sequential presentation of items so that the last item was in FOA by virtue of *recency*. This was then followed by an incidental cue that could be same or different to the last item.

### Methods

#### Participants and stimuli

**Figure 3:**
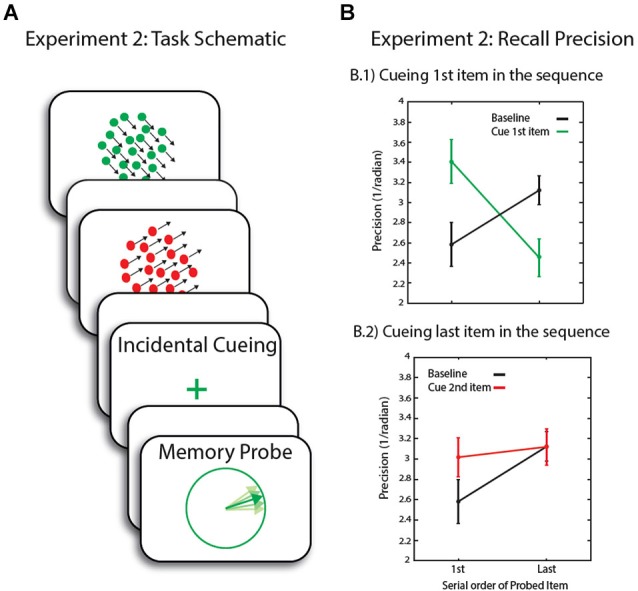
**(A)** Schematic of trial events for Experiment 2. Following a sequential presentation of the memory array, the maintenance period was either blank or included an incidental cue before the presentation of the memory probe. **(B)** Recall precision for motion direction across conditions. Error bars are SEM.

Seventeen healthy individuals (10 male) with an average age of 25 (range: 20–31) participated in Experiment 2. On each trial, two RDKs, consisting ether of red or green moving dots, were now presented sequentially at screen center (Figure 3A), subtending 10° of visual angle. Random dot kinematograms stimulus parameters were otherwise unchanged from Experiment 1.

#### Procedure

Each RDK was presented for 300 ms (+100 ms mask). There was 1000 ms blank delay between the two RDKs. In the ***baseline condition*** (not illustrated in the figure—1/3 of the overall trials), the 2nd RDK was then followed by an unfilled delay of 4.1 s. When cues were presented, the last RDK in the sequence was followed by a delay (1 s) before the presentation of the incidental cue (100 ms), which matched the first RDK in 50% of the trials (1/3 of the overall trials) and the last item for the remaining trials. In this experiment, unlike the previous one, participants had to indicate with a key press the serial position (first or second) of the RDK with the same color as the incidental cue. There was a 3 s delay, which was then followed by the presentation of the probe.

The probe was in the same color as the first RDK on half of the trials and in the remaining trials, it matched the last item in the sequence. This therefore resulted in four experimental conditions with a 2 × 2 design with color of incidental cue as the first factor and probe color as the second factor, both with two levels: color matching the first or second item in the sequence. Participants completed a practice block (30 trials) followed by seven blocks of 30 trials, different conditions randomly intermixed within a block.

### Results

Participant performed the *incidental cueing task* well, with mean accuracy of 95% (SD = 5.3) and mean response time of 878 ms (SD = 200 ms). Trials with incorrect response in the incidental cueing task were excluded from further analysis.

We next investigated the effect of incidental cueing on *precision of recall* of the item in FOA compared to other items in WM. In the *baseline condition*, we obtained the long-established recency effect: the last item was recalled with greater precision than the first. In trials when the first item—presumed not be in the FOA by the end of the sequence—was later cued by the incidental cue, there was a significant interaction between cueing conditions (baseline, cue 1st item, cue last item) and serial order (*F*_(2,32)_ = 9.4, *p* = 0.001).

Comparison of the baseline, no-cue condition to incidentally cueing the 1st item, revealed a significant interaction between cueing condition and serial order (*F*_(1,16)_ = 16.4, *p* = 0.001, Figure 3B.1). This was due to an increase in recall precision for the first item in the sequence (*t*_(16)_ = 2.3, *p* = 0.035)—i.e., for the item now presumed to be in FOA—with a corresponding decrease in precision for the last, normally privileged, item (*t*_(16)_ = 2.8, *p* = 0.012). This replicates our previous findings highlighting that *in the context* of an experiment where a non-privileged item in WM might be probed, it can be retrieved with relatively high precision after focusing attention upon it. In this experiment, unlike the first, we also found a cost in recall of the item that would otherwise be recalled with greater fidelity, here the last one in the sequence.

In trials where the last item in the sequence was cued, there was no significant interaction between cueing condition (cueing last item vs. baseline) and serial order of the probed item. The last item could not be recalled with any greater precision than in the baseline condition. As for the first item, although there was a modest increase in recall precision, this was not significant (*t*_(16)_ = 1.8, *p* = 0.087, Figure 3B.2). It is possible that the small increase in recall precision for the first item was due to rehearsal of items once the last item was cued, before making a response regarding its serial position. Participants might go through the sequence in their mind, resulting in refocusing on the first item before focusing on the last item in the sequence. In fact, response times to incidental cues of the last item in the sequence was marginally longer compared to trials where the 1st item in the sequence is cued (*t*_(16)_ = 1.9, *p* = 0.078).

### Discussion

The results from Experiments 1 and 2 confirm our general hypothesis that in situations where items outside FOA remain *potentially* *behaviorally relevant*, they can be brought into FOA. In Experiment 2, precision of memory decreased for the last item (i.e., the item presumed to be in FOA) in trials where the incidental cue directed attention to the first item, which was now recalled with greater precision than baseline (Figure 3B.1). This simultaneous cost vs. benefit effect is different to that observed in Experiment 1 where recall precision for the defocused item was comparable to baseline, as well as focused condition. However, it is important to note that the effects of Experiment 1 were overall weaker (perhaps due to a much longer delay between encoding and probing of memory than in Experiment 2: 7.2 s vs. 4.1); this could possibly influence the lack of any effect of the de-focused item in the previous experiment.

The effects observed in Experiment 2 might be explained by two different mechanisms. It is possible that the capacity of the FOA is limited, perhaps to just one item, so that in trials where attention shifts to focus on another item in memory, the previously privileged item is displaced from FOA. However, this hypothesis would be inconsistent with some previous observations. Specifically, Rerko and Oberauer (2013) demonstrated an opposite effect for the defocused item than that reported here, with its recall significantly *better* compared to other items in WM for shorter delay periods.

Alternatively, it is possible that the time for which an item might be elevated to FOA by means of some types of cueing is limited. This is supported by EEG findings measuring temporal changes in item representation over the course of a delay period. LaRocque et al. (2013) employed an identical design to that of Lewis-Peacock et al. (2011) with EEG, demonstrating that approximately 1.25 s after cueing the unfocused item, classification accuracy for the initially cued item is decreased to levels comparable to baseline.

## Retro-cueing

If the item in FOA is disrupted by either having attention switch to focus on another item in WM (Experiments 1 and 2) or artificially through TMS to early sensory areas (Zokaei et al., 2014), the item outside FOA can improve to levels comparable to the previously privileged item. However, as discussed previously, this is *dependent on context*: in the previous experiments reported above, non-FOA items remained relevant to the task in hand. Therefore, the question that remains is what happens to the other items in WM when they are rendered largely irrelevant?

Previous literature on this remains controversial (e.g., Landman et al., 2003; Lepsien and Nobre, 2007; Matsukura et al., 2007; Rerko and Oberauer, 2013). Due to differences in methodology comparison across studies to make meaningful general conclusions is difficult. To compare both the behavioral influences of re-focusing attention to other items in WM as well the causal effects of TMS, we conducted two retro-cueing investigations that are closely matched in timing and stimuli to two of our previous studies.

In Experiment 3 we used two successive cues, with the first one being a retro-cue, informative about the nature of the upcoming probe (80% validity). To ensure that participants attended to this cue, the second cue was *either a stay or a switch cue*, with 20% of the trials a switch cue. Importantly, this second cue was 100% valid, so participants knew for sure which item in WM was going to be probed. We aimed to investigate whether people can bring back the previously rendered irrelevant item following a switch trial. This experiment otherwise matched our double incidental cueing paradigm (Experiment 1) with two successive cues in timing and stimuli.

In Experiment 4 we used a similar retro-cue paradigm to that previously employed by Zokaei et al. (2014) (their Experiment 2) with an 80% valid rather than an incidental cue. Transcranial magnetic stimulation was administered during WM maintenance following the presentation of the cue to examine the causal role of early visual areas in maintenance of item in different representational states achieved through retro-cues and importantly its influence on the un-cued items.

## Experiment 3: stay or switch from item in WM that was retro-cued

### Methods

#### Participants

Seventeen healthy individuals (14 Male) with an average age of 28 (range: 22–34) participated in Experiment 3. All had normal or corrected to normal vision and reported normal color vision. Participants provided written consent to the procedure of the experiment, approved by the local ethics committee.

#### Procedure

**Figure 4:**
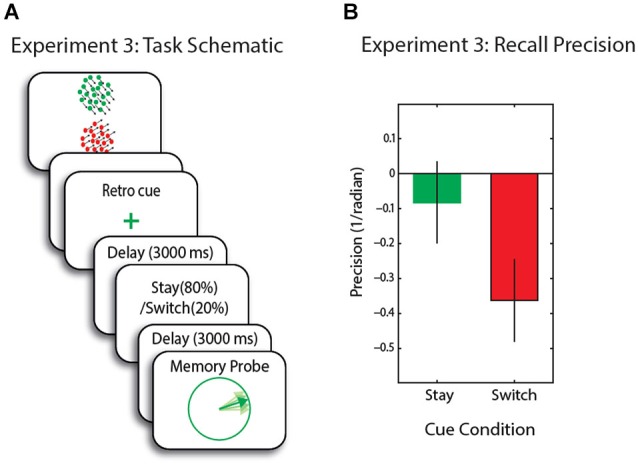
**(A)** Schematic of trial events for Experiment 3. Memory array is followed by a retro-cue with 80% validity regarding the 2nd cue. The second stay/switch cue was 100% valid for the upcoming probe. **(B)** Recall precision for the motion direction across conditions—baseline corrected. Error bars are SEM.

In each trial, similar to Experiment 1, two RDKs were presented above and below the fixation cross (300 ms) followed by a mask (100 ms; see Figure 4A). This was followed by a blank delay of 1000 ms prior to the presentation of the retro-cue, which was in the form of fixation cross color change (100 ms). The fixation cross changed from black either to red or green (50% green) indicating the RDK that was more likely to be the cued later in the trial (80% validity for the next cue). This was then followed by a 3000 ms delay, before presentation of the second cue.

The second cue was 100% valid and was in the form of a word instructing participants to either “stay”, i.e., maintain the same colored RDK as cued before (80% of trials) or “switch”, i.e., switch to the other item WM (20% of trials). Note that in the vast majority of trials, therefore, the first retro-cue could be used to focus attention largely on one item held in WM.

Following a 3000 ms interval the item indicated by the second cue was probed and participants had to adjust the direction of the probe to match that of the cued item. These trials were intermixed with 30 baseline trials with no cueing during the delay but rather a long blank period (7200 ms) before presentation of the probe. Note that in this experiment, we used a switch/stay (second) cue in order to make sure participants attended to the first cue as no response to the that cue was required.

Participants completed a practice block (30 trials) followed by five blocks of 46 trials, different conditions randomly intermixed within a block and were informed of the validity of the cue prior to testing.

### Results

We first applied an ANOVA with cueing condition (valid, no-cue and invalid) as within-subject factors. There was a marginal effect of cue-type; *F*_(2,30)_ = 3.15, *p* = 0.057. Furthermore, recall precision was significantly higher in (80%) stay compared to (20%) switch trials (*t*_(14)_ = 2.7, *p* = 0.0.16; Figure 4B). With respect to the baseline (no-cue) condition, recall precision was not significantly different in stay trials (*t*_(14)_ = 0.8, *p* = 0.4), but significantly decreased in switch trials (*t*_(14)_ = 2.45, *p* = 0.027).

### Discussion

In this experiment the item that was initially retro-cued appeared to be held in a privileged state compared to the other item, which was effectively rendered largely irrelevant, because it was probed on only 20% of trials. These findings are consistent with the hypothesis that in situations where items outside FOA in WM are rendered largely irrelevant by the very low probability of being probed, they cannot subsequently be brought into FOA—at least to the level of precision of cued items—by having attention switched to them. The results are in line with previous experiments using informative cues to direct attention to one/subset of items in WM (e.g., Lepsien and Nobre, 2007; Matsukura et al., 2007; Pertzov et al., 2012). Next we used TMS to area MT+ in an attempt to disrupt the item in FOA.

## Experiment 4: effects of TMS to area MT+

### Methods

#### Participants

Fifteen healthy individuals (8 Male) with average age of 25 (range: 18–32) participated in Experiment 4. All had normal or corrected to normal vision and reported normal color vision. Participants provided written consent to the procedure of the experiment, approved by the local ethics committee.

#### MT+ localization and TMS

A standard approach to MT+ localization using fMRI was applied (Huk et al., 2002). Left hemisphere clusters in the vicinity of MT+ (using anatomical guidelines described by Dumoulin et al., 2000) were identified in the native space of each participant and were overlaid onto their T1-weighted scan for a Brainsight frameless stereotaxy procedure (Rogue Research, Montreal, Canada). The participant’s scalp location of left MT+ was marked on their scalp for subsequent TMS.

Stimulation was delivered via Magtism Rapid^2^ (The Magstim Company, Whitland, Wales, U.K.) using a 70-mm figure-eight coil. The coil handle pointed posteriorly rotated 45º, including a current approximately in the anterior or posterior direction. On each trial, 4 TMS pulses at 20 Hz were applied to left MT+ either at 60% (“high”, effective intensity) or at 24% (“low”, ineffective intensity) of maximum machine output. *Low intensity trials* were used to control for non-specific effects of TMS e.g., acoustic and tactile artifacts.

#### Procedure

**Figure 5:**
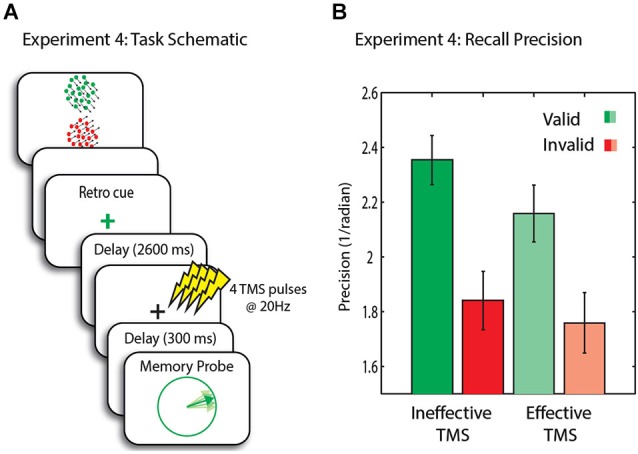
**(A)** Schematic of trial events for Experiment 4. Memory array is followed by a retro-cue with 80% validity. A 20 Hz four pulse TMS train was administered to MT+, followed by a delay and the presentation of the probe. **(B)** Recall precision for the motion direction across conditions. Error bars are SEM.

Similar to Experiments 1 and 3, two RDKs were presented above and below fixation cross (300 ms) followed by a mask (100 ms; see Figure 5A) and blank delay (1000 ms). The fixation cross then changed briefly (similar to Experiment 3), out method of retro-cueing with 80% validity. This was then followed by a delay of 2600 ms before the administration of TMS. The TMS train lasted for 250 ms before presentation of the probe. The probe matched the color of the retro-cue in 80% of the trials (valid trials) while in the remaining 20% it did not (invalid trials). Trial sequence matched closely the timings of Zokaei et al. (2014) (their Experiment 2).

Participants completed a practice block (30 trials) followed by four blocks of 104 trials, different conditions randomly intermixed within a block.

### Results

In this experiment we compared high intensity (effective) TMS to low intensity (ineffective) TMS. Recall precision was significantly higher in valid (i.e., trials in which the probe matched the cue) compared to invalid trials (*t*_(14)_ = 3.2, *p* = 0.006), indicating successful retro-cueing (Figure 5B).

We next assessed whether high vs. low intensity TMS affected recall precision. A two-way ANOVA with factors TMS intensity and trial type yielded a significant main effect of validity (*F*_(1,14)_ = 9.5, *p* = 0.008) and TMS intensity (*F*_(1,14)_ = 4.8, *p* = 0.045). The effect of TMS was due to a significant *decrease* in precision of memory after high intensity TMS on *valid* trials (*t*_(14)_ = 2.4, *p* = 0.029, n.s after correcting for multiple comparisons), importantly with no change on invalid trials (*t*_(14)_ = 0.7, *n.s.*). Thus the privileged item sustained a cost with high intensity TMS. Nevertheless, the behavioral advantage of validity remained, with memory precision being significantly higher for valid compared to invalid trials (*t*_(14)_ = 2.3, *p* = 0.039, n.s after correcting for multiple comparisons).

### Discussion

The results from the TMS experiment are consistent with the hypothesis that in situations where items outside FOA in WM are rendered largely irrelevant by the very low probability of being probed, they cannot be brought into FOA by disrupting the item in FOA with TMS. Here, we used an analogous procedure to that in our previous published study (Zokaei et al., 2014) with the only distinction of having a retro-cue rather than an incidental cue. To the best of our knowledge, that report is one of the first to investigate the causal role of early visual areas in maintenance of the retro-cued item in WM demonstrating that the maintenance of the cued item, in line with previous imaging studies (Lepsien and Nobre, 2007; Harrison and Tong, 2009; Nelissen et al., 2013), relies to some extent on visual areas involved in its perception (Zokaei et al., 2014).

In the current experiment, TMS to MT+ did not have an effect on the other item retained in WM, unlike the previously observed result with an incidental cue (Zokaei et al., 2014). In the study reported here, there was no improvement in recall precision for the other item in memory presumably because high quality information regarding this item was lost because of the extremely low (20%) probability of being probed. It is, however, important to note that the smaller number of invalid relative to valid trials may also explain the lack of TMS effect on the invalid condition.

## General discussion

The studies reported here sought to investigate the flexibility of representational states in WM: whether retained information can be flexibly moved in and out of the privileged state of FOA. Across four experiments, we explicitly and implicitly manipulated the state of two items in WM using different methods in order to place one in the FOA. Taken together, our results suggest that although the item in FOA is represented with higher recall precision compared to the non-privileged items in all experiments, the nature of the other items in WM crucially depends on the relevance of these items to the WM task. The *context* in which people are required to hold more than one item in WM makes a difference to how flexibly they can switch attention to non-focused items and improve their precision of recall. Also interesting to note is that the item in FOA was never recalled with greater precision than uncued items in the baseline condition. This suggests that the benefit conferred by the FOA is not necessarily due to straightforward increase in the precision with which the item is maintained compared to all other items. Indeed, this result fits with those of Gorgoraptis et al. (2011) in which recall precision of the last item in the sequence (i.e., the item in FOA) was similar to the precision of items when presented simultaneously regardless of memory set size. The implication is therefore that the FOA benefit may also depend on the context of the experiment.

In Experiment 1 we used either one or two consecutive incidental cues to explicitly bring the cued item into FOA: by requiring participants to make an action regarding a feature (in this case, location) of one of the retained items during WM delay period. The consequence of such incidental cueing is that this item, if probed later (congruent condition), will be recalled with higher precision than if the alternative item is probed (incongruent condition) (Zokaei et al., 2014). In Experiment 2 we used sequential presentation of the memory array followed by an incidental cue (in this case regarding, serial order), with the assumption that the last item in the sequence was in the privileged state by virtue of recency. Our working hypothesis, based on previous published studies discussed in the Section Introduction, is that the last cue in both these experiments places the cued item in the FOA. But importantly it carries no information regarding the relevance of the cued item for the upcoming WM task.

The results from these two studies demonstrated that in fact in situations where the item outside FOA *remains potentially relevant* to the task in hand, it can be brought back into the privileged state. Theoretically, these findings are in line with predictions made by the three-embedded components theory (Oberauer, 2002, 2009; Rerko and Oberauer, 2013) where no permanent detrimental effect on the other items in WM is predicted. In the context of this theoretical framework, the decrease in recall precision of the unfocused items might be due to direct interference *from the item in FOA* (e.g., Pertzov et al., 2012). Thus abolishing such interference, either through disruptive effects of TMS (Zokaei et al., 2014) or by re-focusing attention on non-privileged items (as in Experiments 1 and 2 here), can increase their WM precision to levels comparable to baseline.

If the items outside FOA are indeed maintained and can be brought back into FOA, then what are the mechanisms underlying their retention? A few studies have demonstrated a role of the hippocampus in maintenance of items outside FOA, with their retrieval accompanied by activation in MTL (Oztekin et al., 2009, 2010; Nee and Jonides, 2011). Consistent with such observations, patients with hippocampal sclerosis have impaired WM performance only for items presented earlier in the sequence, that is items outside the FOA (López-Frutos et al., 2014). Alternatively, it has been suggested that the maintenance of unfocused items is supported either through sustained rapid short-term synaptic plasticity (Mongillo et al., 2008; Buonomano and Maass, 2009; Stokes et al., 2013) or sustained neuronal firing in non-sensory regions (Fuster and Alexander, 1971; Goldman-Rakic, 1995). However, support for each of these accounts is sparse and the brain mechanisms by which an item is brought into FOA have yet to be elucidated.

Unlike in the first two experiments, in Experiments 3 and 4 we manipulated representational states using retro-cues, i.e., cues that appear to focus attention on the most relevant item in WM for the upcoming probe. In Experiment 3, we used two consecutive cues: the first one was a retro-cue, informing participants on the likely nature of the second cue with 80% validity. The second cue was 100% valid for the WM probe. Recall precision for the invalidly cued WM item (i.e., the item probed on only 20% of trials) was significantly lower compared to the validly cued one, despite having attention switch to this item with a second 100% valid retro-cue. Therefore, the irrelevant item in WM following a retro-cue could not be brought back into FOA, despite having attention focus on it via the second retro-cue. Thus some information was, to some extent, irretrievably lost from WM following the initial retro-cue.

In Experiment 4, the precision of memory for the other/irrelevant items did not improve following disruptive effects of TMS to the item in FOA, unlike previous studies using incidental cueing or sequential presentation of items (Zokaei et al., 2014). However, while the probability of being probed regarding the invalid item was extremely low (20%) in the TMS study we report here, in the incidental cueing study of Zokaei et al. (2014), there was a 50% probability of being probed on the uncued item.

In that report, the improvement of memory for the non-FOA item in WM following TMS was described in terms of weakening of the interference from the item in FOA on the non-privileged item. According to this hypothesis, TMS abolished the advantage of the item in FOA, leaving “baseline” WM performance intact and resulting in normalized performance for all items in WM. In accordance with such hypothesis, in the retro-cueing TMS study reported here (Experiment 4), “baseline” performance for the non-FOA items was decreased due to validity of the cue, prior to the administration of TMS. Abolishing the interference of item in the privileged state would therefore not be expected to improve the quality of the memory for the non-FOA item since it is maintained with low precision to begin with.

Findings from previous studies are not all consistent. Few have reported a cueing advantage (Lepsien and Nobre, 2007; Matsukura et al., 2007; Pertzov et al., 2012) while others have failed to observe one (Landman et al., 2003; Rerko and Oberauer, 2013). Current findings however can potentially explain such discrepancy in the literature if one considers the amount of information conveyed by the retro-cue regarding the likelihood of being probed on uncued items. In experiments that demonstrate a cueing advantage, the cue was valid, i.e., the cued item was later probed in majority of trials. On the other hand, in studies that have failed to report any effect of cueing, the cue was only 50% valid and hence no different to a no cue condition. In addition, some studies using two consecutive cues have no method of confirming whether participants attended to the cues or not (e.g., Landman et al., 2003; Rerko and Oberauer, 2013), unlike the methods used here. Together these factors may well contribute to the inconsistency in the literature.

Our findings, in light of previous literature, suggest that the magnitude of cueing is dependent on the amount of information conveyed by the cue—the *context* in which WM holds items. Future research might aim to directly test this hypothesis by varying the validity of the cue systematically, although as the frequency of invalid cues is reduced the number of trials required to generate sufficient data would increase massively. Furthermore, current advances in modeling the sources of error associated with a specific pattern of performance may shed light on possible mechanisms that an item gains its privileged state (Bays et al., 2009). Similar findings with memory set sizes larger than two are also essential in building a comprehensive understanding of flexibility of representational states in WM.

The findings reported here also have important implications for both theoretical and neural computational models of WM. Although some have proposed dynamic representational states of items during WM retention (Fujisawa et al., 2008; Barak et al., 2010; Pascanu and Jaeger, 2011; Stokes et al., 2013), most models assume static representations (Seung and Sompolinsky, 1993; Lisman et al., 1998; Compte et al., 2000; Durstewitz et al., 2000; Mongillo et al., 2008; Dempere-Marco et al., 2012; Wei et al., 2012). Our results provide support for remarkable flexibility of representations in WM *dependent upon context*, and its close relationship to directed attention. Moreover, state-models of WM do not often make the distinction between other/non-privileged items in WM in different contexts, assuming a similar fate for all these items irrespective of how they have achieved their state. Our results provide strong empirical support for differential effects of cue validity—context of the WM task—on the nature of items outside FOA. Given the compelling evidence provided here, both computational and theoretical models of WM might profitably take into account dynamic representational states in WM when several items are retained.

## Conflict of interest statement

The authors declare that the research was conducted in the absence of any commercial or financial relationships that could be construed as a potential conflict of interest.
